# Glycemic outcomes of Advanced Hybrid Closed Loop system in children and adolescents with Type 1 Diabetes, previously treated with Multiple Daily Injections (MiniMed 780G system in T1D individuals, previously treated with MDI)

**DOI:** 10.1186/s12902-022-00996-7

**Published:** 2022-03-29

**Authors:** Goran Petrovski, Fawziya Al Khalaf, Judith Campbell, Emma Day, Douha Almajaly, Khalid Hussain, Maheen Pasha, Fareeda Umer, Manar Hamdan, Amel Khalifa

**Affiliations:** Department of Pediatric Medicine, Division of Endocrinology and Diabetes, Sidra Medicine, PO Box 26999, HB 6E 219, Al Luqta Street, Education City North Campus, Doha, Qatar

**Keywords:** Type 1 diabetes, Continuous subcutaneous insulin infusion, Continuous glucose monitoring, Diabetes education, Closed-loop systems

## Abstract

**Background:**

The objective of this study was to evaluate the glycemic outcomes in children and adolescents with Type 1 Diabetes (T1D) previously treated with Multiple Daily Injections (MDI) using a structured initiation protocol for the Advanced Hybrid Closed Loop (AHCL) Minimed 780G insulin pump system.

**Methods:**

In this prospective open label single-arm, single-center, clinical investigation, we recruited children and adolescents (aged 7–17 years) with T1D on MDI therapy and HbA1c below 12.5%. All participants followed a 10-day structured initiation protocol which included 4 steps: step 1: AHCL system assessment; step 2: AHCL system training; step 3: Sensor augmented pump therapy (SAP) for 3 days; step 4: AHCL system use for 12 weeks, successfully completing the training from MDI to AHCL in 10 days. The primary outcome of the study was the change in the time spent in the target in range (TIR) of 70–180 mg/dl and HbA1c from baseline (MDI + CGM, 1 week) to study phase (AHCL, 12 weeks). The paired student t-test was used for statistical analysis and a value < 0.05 was considered statistically significant.

**Results:**

Thirty-four participants were recruited and all completed the 12 weeks study. TIR increased from 42.1 ± 18.7% at baseline to 78.8 ± 6.1% in the study phase (*p* < 0.001). HbA1c decreased from 8.6 ± 1.7% (70 ± 18.6 mmol/mol) at baseline, to 6.5 ± 0.7% (48 ± 7.7 mmol/mol) at the end of the study (*p* = 0.001). No episodes of severe hypoglycemia or DKA were reported.

**Conclusion:**

Children and adolescents with T1D on MDI therapy who initiated the AHCL system following a 10-days structured protocol achieved the internationally recommended goals of glycemic control with TIR > 70% and a HbA1c of < 7%.

## Background

A high proportion of people with Type 1 Diabetes (T1D) across all age-groups do not achieve glycemic targets [1], implying that new approaches and therapies are required to optimize metabolic control. Diabetes management which avoids hypoglycemia in T1D requires frequent decisions in insulin dosing that impact glucose levels and insulin sensitivity, leading to day-to-day variability of glucose levels and insulin requirements [2], resulting in a high burden of diabetes management.

Hybrid Closed-Loop (HCL) systems use specific control algorithms [3] which automate basal insulin delivery, based on glucose sensor values [3, 4] for preventing both hypoglycemia and hyperglycemia [5, 6]. Several studies have shown that HCL systems improves HbA1c and time in range (TIR) in children [7], adolescents and adults [8] with T1D, while reducing hypoglycemia and lowering HbA1c compared to gold-standard insulin therapy [9]. However, some centers reported continuing challenges with the previous HCL system (MiniMed 670G), in youths with higher HbA1c compared with youths with lower HbA1c, mainly related to the increased workload required to use the MiniMed 670G system [10].

The new generation of HCL systems now have additional features of automated bolus insulin correction for high glucose levels. This further improves glycemic control and reduces the health burden for people with T1D.

These systems have the potential to improve glycemic outcomes across a broad age range and in people with different types of diabetes [11], being of benefit to adolescents, young adults [12] and older adults with variable HbA1c concentrations at baseline [13].

Reduction in the risk of hypoglycemia with continued improvement in glucose control was also demonstrated across a wide age range of patients with sub-optimally controlled T1D [14].

One of the latest HCL systems is the MiniMed 780G system (Medtronic, Northridge, CA, USA), which was commercialized in October 2020 in selected countries. This system automatically delivers basal insulin with a customizable glucose target in addition delivering an automated bolus correction, as required. A pre-commercial trial [15] has shown a reduction in time in hyperglycemia, without increasing hypoglycemia, compared to the MiniMed 670G HCL system. The pivotal trial data [16] demonstrated that the system was safe and significantly improved HbA1c and sensor glucose (SG) levels in adolescents and adults with T1D and provided rapid glycemic improvements in people with T1D with pre-existing good glycemic control [17].

The above-mentioned studies have evaluated the Advanced Hybrid Closed Loop (AHCL) system use in people with T1D, previously treated with insulin pump therapy. To the best of our knowledge, this is the first clinical experience on the AHCL MiniMed 780G system in people with T1D, previously treated with Multiple Daily Injections (MDI), without prior pump experience.

The objective of this study was to evaluate the glycemic outcome using a structured initiation protocol for AHCL Minimed 780G system in children and adolescents with T1D on MDI therapy.

## Methods

### Study design and participants

In this prospective, single center, single arm, intervention study on AHCL MiniMed 780G system (Medtronic, Northridge, CA USA), we recruited children and adolescents (aged 7–17 years) attending the diabetes clinics at Sidra Medicine (Doha, Qatar), on a first come, first served basis. Patients were eligible if they had T1D [18] with HbA1c less than 12.5%; had been using MDI for at least one year, with or without Real Time Continuous Glucose Monitoring (rtCGM) or Intermittent Scanning Continuous Glucose Monitoring (isCGM), with no prior pump experience. Exclusion criteria included total daily insulin dose less than 8 units and any episode of Diabetic Ketoacidosis (DKA) in the last 6 months.

The study was approved by the institutional review board at Sidra Medicine in Doha and national ethics committee at Ministry of public health in Qatar and all participants, and their guardians signed a written informed assent/consent before the start of study-related procedures.

### Procedures

Individuals were recruited at regular clinic visits on a first come first served basis, according to a clinical pre-pump assessment on the following aspects: frequency of Self-Monitoring of Blood Glucose (SMBG) measurements, minimum of 3 measurements per day, for patients using CGM devices the assessment was related to appropriate calibrations (if any) and/or number of scans, knowledge of carbohydrate counting, insulin dose adjustments based on carbohydrate intake and glucose level, the ability to identify changes in insulin requirements due to physical activity and sick days, ability to recognize, troubleshoot and manage hyperglycemia and hypoglycemia.

Funds for devices were secured through the medical insurance or self-funding coverage and through donations made by Qatar Diabetes Association for individuals who could not afford the pump therapy. There were no rejections due to funding restraints.

**Fig. 1:**
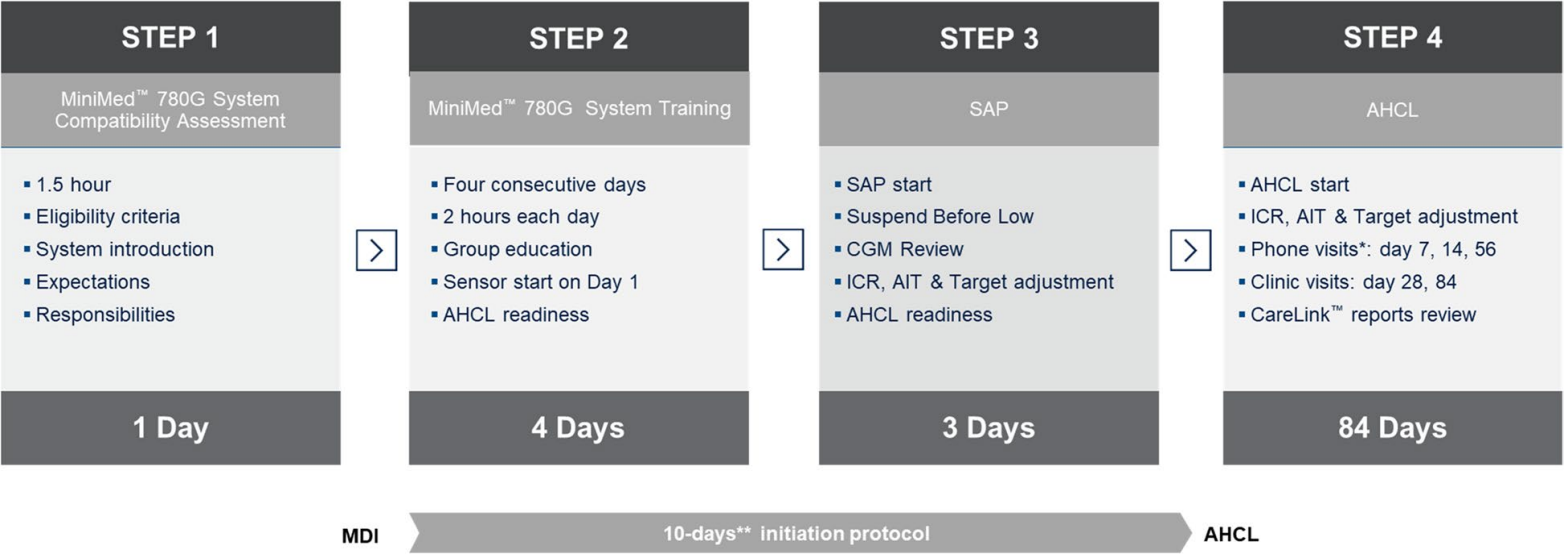
Steps of 10 Days Initiation Protocol. * On day 4 CareLink reports were reviewed, and the participant is contacted by phone if deemed necessary. ** Including 3 days between steps 2 and 3. CGM, continuous glucose monitoring; SAP, Sensor Augmented Pump; AHCL, Advanced Hybrid Closed Loop System; ICR, Insulin to Carbohydrate Ration; AIT, Active Insulin Time

The initiation protocol consisted of four main stages (Fig. 1):

- Step 1: AHCL system introduction. Interested individuals attended one introduction session of one hour (groups of 5–8), where the MiniMed 780G system was described. Individuals’ responsibilities and commitments (bolus insulin before meal, calibrating the system 2–3 times per day, responding to alerts and alarms, attending all training sessions), system expectations (improvements in glycemic control, TIR concept) and CareLink mobile application were also discussed. The importance of the SMBG was emphasized in those individuals using CGM device without calibration.
- Step 2: AHCL system training. Two to four individuals and their parents/guardians attended group training sessions. The program included four sessions of two hours on four consecutive days.
  - Day 1: Technical set-up of transmitter, linked BG meter, MiniMed Mobile App and CareLink Connect application, pump navigation, menus and icons, understanding CGM graph, education in sensor calibration and sensor insertion.
  - Day 2: Bolus wizard set-up, basal rate settings, Operational modes; Manual Mode & Smartguard, AHCL exits, AHCL readiness, infusion set and reservoir change.
  - Day 3: Theory sessions; hypoglycemia, hyperglycemia and ketones, exercise, travel management, and temporary target.
  - Day 4: Download report review and evaluation compliance, revision and review by diabetes educator of participants’ ability to manage the AHCL system.
    Each session was provided by diabetes educators from 12 to 2 pm. CGM was initiated the first day of the training, for education and observational purposes and for baseline data collection (no insulin delivery by the pump). Timing of the long-acting insulin injection was moved two hours ahead each day during training sessions, to reach 12 pm the day before the AHCL system was initiated as Sensor Augmented Pump (SAP), to avoid the use of temporary basal feature at insulin pump initiation. The participants used the MiniMed 780G as a rtCGM (no insulin delivery by pump) for 3 days to gain more experience and confidence with the sensor, before proceeding to Step 3. The importance use of SMBG for calibration was emphasized in those individuals using prior CGM device without calibration and for those initiating CGM.
- Step 3: SAP Initiation. Participants initiated the use of the AHCL system as SAP with suspend before low feature for 72 h to allow the algorithm to collect insulin utilization and CGM data to establish personalized parameters for the AHCL initiation. Sidra’s validated protocol for SAP initiation, as previously described [19], with review of CGM data at the end of step 2 was used: in short, the protocol inputs the current insulin program (MDI), calculates a 10 to 20% reduction of total daily dose, with a 40/60 basal/bolus distribution in four or five basal rates. Insulin to Carbohydrate Ratio (ICR) settings utilizes the formula of 300–450/Total Daily dose (TDD) and, the formula of 90–110/TDD (mmol/l) with two Correction Factors (CF) settings, the nighttime CF set 10–20% higher than the daytime CF. Active insulin Time (AIT) is set on 3 h, suspend before low feature is turned on with a threshold of 55–70 mg/dl (3.0–3.8 mmol/l) and glucose target range from 90–130 mg/dl (5.0 to 7.2 mmol/l).
- Step 4: AHCL Initiation and Follow up. AHCL was initiated as follows: age > 12 years, algorithm glucose target 100 (5.5 mmol/l), AIT 2 h; age < 12 years, algorithm glucose target 100 (5.5 mmol/L) if TBL < 3%, 110 mg/dl (6.1 mmol/L) if TBL < 5% and 120 mg/dl (mmol/l) if TBL > 6%, AIT 2–4 h; and automated bolus correction ON). AHCL was used continuously for 12 weeks, i.e. in case of AHCL exit, the participants were instructed to perform the actions recommended by the pump to re-enter AHCL. Follow up visits were scheduled in the clinic on weeks 2 and 12, and by phone on weeks 1, 4 and 8 after initiating AHCL and the systems settings were re-assessed and adjusted if needed per clinical judgement.

No restriction in dietary intake or daily activities were advised during the study. Clinical and technical support was available at all times with text messaging and phone calls during the study. Standard local hypoglycemia and hyperglycemia treatment guidelines were followed.

HbA1c was measured with point of care DCA Vantage Analyzer (Siemens, Erlangen, Germany) at baseline and at the end of the study.

### Outcomes

The primary outcome was the TIR change and HbA1c from baseline (MDI + CGM, 1 week) to study phase (AHCL,12 weeks). The secondary outcomes included changes in time below range (TBR), time above range (TAR), insulin delivery (TDD, basal, bolus and auto-correction distribution), AHCL system settings (ICR, AIT, glucose target) and system usage (sensor wear time, time in AHCL). Time spent in the different glycemic ranges was also evaluated for different time periods: MDI + CGM (1 week), SAP (3 days), AHCL (week 1, week 2, week 3 and 4, week 5–8, week 9–12).

Safety outcomes were measured by recording episodes of severe hypoglycemia and/or Diabetic Ketoacidosis (defined by American Diabetes Association), both requiring medical attention.

Treatment satisfaction was assessed using Diabetes Treatment Satisfaction Questionnaire (DTSQ)s Parents and Teens [20], which assesses the changes in patient and caregiver satisfaction related to therapy modifications and is also useful for comparing the level of satisfaction in patients using different treatment strategies. The DTSQ is a 14-item measure for parents and 12-item measure for children. All items are rated from 0 (very unsatisfied) to 6 (very satisfied), and the range of the total score is 0 to 84 (for parents) and 0 to 72 (for children, with higher scores indicating better satisfaction). Permission to use both parent and participant DTSQ was granted by the authors.

### Statistical analysis

This study was exploratory, and no power calculations were required. We planned to recruit 34 participants, aiming for 31 participants to complete the study to allow for dropouts (anticipated dropout rate of 5–10% based on the investigators’ experience and expectation).

The statistical analysis plan followed the completion of the last patient’s last visit but before the final dataset was reviewed and analyzed.

Analysis was performed for the entire study population. Insulin and CGM data were collected from CareLink Therapy Management Software during the study. Values are presented as mean (SD) or as a percentage for each study period. The paired student t-test or paired Wilcoxon test (in case of non-normality) was used in the study. A value < 0.05 was considered statistically significant. Statistical analyses were performed using Statistica 12 (Stat Soft, Tulsa, OK, USA).

## Results

**Fig. 2:**
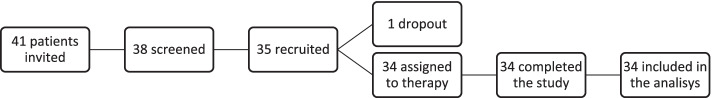
Trial profile

Between October 25, 2020, and February 5, 2021, 41 individuals were invited to take part in the study, however three declined due personal reasons. Three of thirty-eight individuals who were screened did not meet the patients’ responsibilities and requirements for the system (carb counting and SMBG ≥ 3 times per day, or as required for CGM calibration) (Fig. 2). One participant withdrew during baseline (Step 2) due to social and family issues. The protocol was implemented in 34 participants, and they all completed the planned 12 weeks on AHCL use (Fig. 2). Baseline characteristics are shown in Table 1. Eighteen participants (53%) were using SMBG with glucometer and had no prior diabetes technology experience (CGM use).

**Table 1:** Study participant’s characteristics at baseline

	**Participants** ***N* = 34**
**Age, years**	12.5 ± 3.7
**Male, n (%)**	16 (47%)
**Female, n (%)**	18 (53%)
**Weight, kg**	48.2 ± 16.7
**BMI, kg/m2**	20.4 ± 3.8
**BMI, percentile**	76.8 ± 10.4
**Duration of diabetes, years**	4.3 ± 2.9
**TDD, U/(kg/d)**	0.9 ± 0.3
**HbA1c, mmol/mol**	70 ± 18.6
**HbA1c, %**	8.6 ± 1.7
**Sensor use, n (%)**	
** RT CGM**	6 (18%)
** isCGM**	10 (29%)
**No Sensor**	18 (53%)

All values are shown as mean ± SD, except for gender and sensor use

*BMI* Body Mass Index, *TDD* Total daily dose of insulin, *SD* Standard Deviation, *RT-CGM* Real time continuous glucose monitoring (Dexcom G5/Guardian Connect), *isCGM* Intermittent continuous glucose monitoring (Freestyle libre)

### HbA1c and time in ranges

HbA1c decreased from 8.6 ± 1.7% (70 ± 18.6 mmol/mol) at baseline, to 6.5 ± 0.7% (48 ± 7.7 mmol/mol) at the end of the study (*p* = 0.001) and TIR (70-180 mg/dL) increased from 42.1 ± 18.7% at baseline to 78.8 ± 6.1% in study phase (*p* < 0.001) (Table 2). TBR did not change while TAR decreased significantly (Table 2). Mean SG decreased from 198 ± 38 mg/dL to 138 ± 12 mg/dL (*p* = 0.001). The majority of the patients reached the consensus glycemic goals with 79% of participants reaching HbA1c < 7% and 74% of participants reaching TIR 70%.

**Table 2:** Glucose control, HbA1c, insulin delivered during baseline and study phase

	**Baseline** **MDI + CGM, 1 week**	**Study** **AHCL, 12 weeks**	***P***
**HbA1c, (mmol/mol)**	70 ± 18.6	48 ± 7.7	0.001
**HbA1c, (%)**	8.6 ± 1.7	6.5 ± 0.7	0.001
Sensor glucose (mg/dL)	198 ± 38	138 ± 12	0.001
**Percent of sensor glucose values in range**			
** < 54 mg/dL**	0.8 ± 0.7	0.5 ± 0.4	0.008
** 54–70 mg/dL**	2.4 ± 2.1	2.3 ± 1.2	0.624
** 70–180 mg/dL**	42.1 ± 18.7	78.8 ± 6.1	0.001
**180–250 mg/dL**	28.1 ± 9.7	13.4 ± 5.1	0.001
** > 250 mg/dL**	26.6 ± 16.2	5.0 ± 2.2	0.001
** TDD, U/(kg/d)**	0.9 ± 0.3	1.1 ± 0.3	0.008
** Basal insulin, as % of TDD**	38.6 ± 7.5	31.6 ± 6.1	0.001
** Weight, kg**	48.2 ± 18.5	49.3 ± 17.2	0.800

All values are shown as mean ± SD

*MDI* Multiple Daily Injections, *SG* Sensor glucose, *TDD* Total daily dose of insulin

### Time in ranges evolution over time

**Fig. 3:**
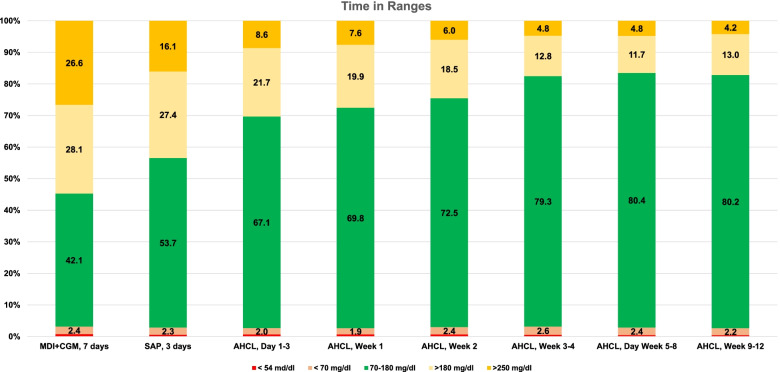
Time in Ranges during Baseline, SAP and AHCL periods. Values are shown as percentage spent in ranges during the interval. Glucose values < 54 mg/dl are not shown on the graph: 0.8% in MDI + CGM, 0.6% in SAP and AHCL Day 1–3, 0.7% in AHCL Week 1, 0.6% in AHCL Week 2, 0.5% in AHCL Week 3 and 4 and AHCL Week 5–8, and 0.4% in AHCL Week 9–12. TIR changes: MDI + CGM to SAP (*p* = 0.005), SAP to AHCL Week 1 (*p* = 0.003), AHCL Week 2 to AHCL Weeks 3 and 4 (*p* = 0.010), AHCL Weeks 3 and 4 to AHCL Weeks 5–8 (*p* = 0.234) and AHCL Weeks 5–8 to AHCL Weeks 9–12 (*p* = 0.147). MDI, multiple daily injections; CGM, continuous glucose monitoring; SAP, Sensor Augmented Pump; AHCL, Advanced Hybrid Closed Loop System

Comparing different study periods, as shown in Fig. 3, TIR increased from MDI + CGM to SAP (*p* = 0.005), SAP to AHCL Week 1 (*p* = 0.003) and AHCL Week 2 to AHCL Weeks 3 and 4 (*p* = 0.010). TIR unchanged from AHCL Weeks 3 and 4 to AHCL Weeks 5–8 (*p* = 0.234) and AHCL Weeks 5–8 to AHCL Weeks 9–12 (*p* = 0.147). TAR (> 180 mg/dL) decreased from a mean of 55.7% at MDI + CGM to a mean of 17.2% at weeks 9–12 of AHCL (*p* = 0.001).

### TDD and insulin delivery distribution

TDD increased by a mean of 0.2 Unit/kg (p = 0.008) at the end of the study, compared to the baseline and basal insulin delivery decreased by a mean of 7% for the same period, *p* < 0.001 (Table 2). TDD during the third month of AHCL use was distributed between auto basal (30%), auto bolus (14%), manual bolus (56%).

### AHCL system usability

After initiating AHCL, the participants used the sensor for a mean of 94.5 ± 7.2 of the time and spent a mean of 91.2 ± 4.6% in AHCL. The number of AHCL exits per patient per week was 0.7 ± 0.8 in the first two weeks and 0.5 ± 0.7 during the third month of AHCL use (*p* = 0.221), as shown in Table 3. The mean number of SMBG significantly decreased from the first two weeks in AHCL use to the third month of AHCL use, *p* < 0.001 (Table 3). The number of meals increased in the third month of AHCL use from 4.4 to 5.2 (*p *= 0.042) compared to the first two weeks of AHCL initiation (Table 3). Infusion set and reservoir were changed on the regular basis every 2–3 days and number of carbohydrate intake did not differ from the beginning of AHCL use to the end of the study (Table 3).

**Table 3:** AHCL system characteristics during the study

	**First two weeks in AHCL**	**Third Month in AHCLS**	***P***
**Sensor wear, %**	92.7 ± 10.1	95.8 ± 5.3	0.118
**AHCL usage, %**	88.4 ± 5.7	91.8 ± 3.2	0.003
**SMBG, n per day**	5.5 ± 1.2	3.4 ± 0.9	0.001
**Set change, n of days**	2.5 ± 0.5	2.5 ± 0.7	1.000
**Res change, n of days**	2.8 ± 0.6	2.7 ± 0.6	0.494
**Meals, n per day**	4.4 ± 1.2	5.2 ± 1.9	0.042
**Carbs, gr per day**	209 ± 78	213 ± 79	0.834
**ICR, gr**	11.3 ± 6.9	8.5 ± 3.8	0.042
**AIT, h**	2.6 ± 0.7	2.5 ± 0.6	0.523
**Exit from AHCL per patient per week**	0.7 ± 0.8	0.5 ± 0.7	0.221

Values are shown as mean ± SD. *Values shown as mean and SD

*n* Number, *AIT* Active Insulin Time, *Res* Reservoir, *Carbs* Carbohydrates, *ICR* Insulin to carb ratio, *SG* Sensor glucose, *Max* maximum, *AHCL* Advanced Hybrid Closed Loop System

ICR during the study phase were made more aggressive regardless of the meal period compared with baseline and decreased from 14.8 ± 7.0 g on MDI + CGM to 13.1 ± 7.3 g on SAP (*p* = 0.350). ICR further decreased to 11.3 ± 6.9 g in the first two week of AHCL use (*p* = 0.041). This trend of strengthening the ICR to 8.5 ± 3.8 g was also noticed in the third month of AHCL use (*p* = 0.001) (Table 3).

**Fig. 4:**
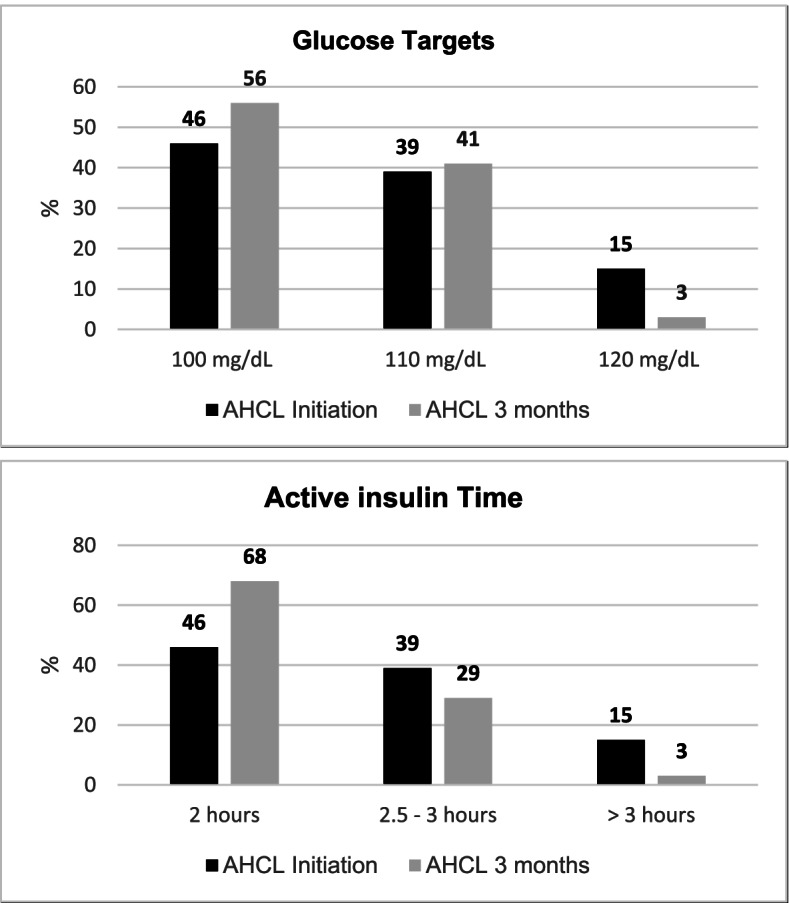
Glucose targets and Active insulin time during AHCL system at baseline and at end of the study. Values are shown as % of participants. AHCL, Advanced Hybrid Closed Loop System

Forty-six percent of the participants initiated the AHCL system with a glucose target of 100 mg/dl and an AIT of 2 h and 15% of individuals with algorithm glucose target of 120 mg/dl and AIT > 3 h (Fig. 4). More than 90% of participants finished the study with algorithm glucose targets of 100 mg/dl or 110 mg/dl with AIT from 2–3 h (Fig. 4).

### DTSQ analysis

The average score of participants’ DTSQ in participants increased from 3.6 ± 0.6 at baseline to 4.6 ± 0.8 at the end of the study (*p* = 0.001). Similar results were found in parents’ DTSQ average score of 3.5 ± 0.6 at baseline and 4.8 ± 0.9 at the end of the study (*p* = 0.001).

### Safety

No serious adverse events, episodes of severe hypoglycemia or hyperglycemia with ketosis were reported. Skin irritations related to sensor use occurred in four participants. Three participants had mild respiratory tract infections. All reported adverse events were resolved without sequelae.

## Discussion

In this prospective open label single-arm, single-center, clinical investigation, we demonstrated that children and adolescent with T1D on MDI can improve their glycemic control in a safe manner using a 10-day initiation protocol. Beneficial effects on glycemic outcomes included increased TIR (70–180 mg/dl), decreased HbA1c levels, reduced time above the range without compromising hypoglycemic events, and decreased mean glucose concentration. 87% of invited patients consented to the study and no attrition was observed during the 3-month follow-up. The patients will be monitored to determine long-term persistence and outcomes.

The high AHCL engagement in our study was demonstrated by high sensor usage and percent time in automated insulin delivery (AHCL use). The sensor use of 98% was significantly higher compared to 86% in a study with same device (15), but similar to 97% in children using AHCL with a different algorithm [21] and 90% in people with previous pump experience [14]. The high sensor usage and high AHCL use in our study can be explained with the algorithm modification in Minimed 780G, like automated bolus corrections, increased time to exit from AHCL usage, increased time of Umax and Umin insulin delivery.

Participants’ high engagement and no attrition can be an indicator of their motivation and satisfaction with the AHCL system in improving their glycemic control.

In our study, the mean TIR (70-180 mg/dL) achieved was 79%, which is significantly higher than 67% achieved with the same device, but without optimization of AIT and glucose target [15] and similar to a mean of 80% achieved with same device in adolescents and adults with previous SAP experience [17]. In a subgroup of participants of AHCL MiniMed Pivotal study [16], where the algorithm glucose target was set to 100 mg/dl and AIT to 2 h, TIR (70–180 mg/dl) increased to 78.8%, which is similar to our findings where more than 60% of participants had same settings. TIR (70–180 mg/dl) results achieved in our study are noticeably higher by more than 20% than those results of the same age [21], adolescents and young adults [14], both using different closed loop algorithms. Time in range, time below range, and time above range observed in our study, all achieved the desired clinical targets for CGM data interpretation [22]. We therefore presume that combining the MiniMed 780G algorithm with the structured initiation program are the core underlying reasons for the successful outcomes.

These clinical outcomes coincided with the high sensor and AHCL use. The new algorithm with automated correction boluses and more stringent pump settings (AIT of 2–3 h and algorithm glucose target of 100 mg/dl (5.6 mmol/l) and 110 mg/dl (6.1 mmol/l)) allowed more robust personalization of the therapy, which lead to improved TIR and higher percentage of time spent in AHCL. Less exits from AHCL use compared to previous Minimed 670G HCL system, likely plays a significant role in the success of the current AHCL system. The parental/guardian involvement and supervision of the children, the support and follow up by the diabetes team and our specific initiation protocol might be additional factors for these outcomes. The significant TIR improvement reaching more than 70% after only 7 days of AHCL use which indicates the rapid adaptation on the algorithm to the specific needs of the individual, as well the effectiveness of our structured initiation protocol. TIR continued to improve over time until reaching a plateau of 80% after 2 months of AHCL use.

The reduction in HbA1c, by a mean of 2.1% (23 mmol/mol), observed in our study are more than 1.6% reduction with a previous HCL (MiniMed 670G) system [19], greater than 0.6% in sub- optimally controlled patients with T1D [14] and 0.6% in children with T1D [21], both using different AHCL algorithms. Moreover, 79% of the participants reached HbA1c < 7.0% (53 mmol/mol) at the end of the study, which is the target established by the ADA and ISPAD guidelines for glycemic control in children [23, 24]. This is also significantly higher compared to the AHCL MiniMed pivotal trial [16].

Our protocol is based on using the system in open loop for only 72 h before initiating AHCL, which is sufficient time for the algorithm to generate personal automated insulin delivery patterns. This period of 72 h was previously reported with HCL MiniMed 670G, as a novel approach in HCL initiation [19]. The protocol is designed for HCL and AHCL (Medtronic) systems based on their specifics (algorithm, target glucose level, software) and it might need modification and adaptation for other closed loop systems.

In our study, the short period of both pump training and SAP use, did not impact the glycemic control and safety of the participants; on the contrary, the superior clinical outcomes that were achieved in mean HbA1c of 6.5% and in mean TIR (70–180 mg/dL) of 79%.

Additional insight from our experience found that modifying ICR by almost 40% by increasing the meal bolus dose from baseline is necessary when initiating individuals on AHCL system from MDI regimens. This is significantly higher than the 20% increase required with a similar protocol with the previous MiniMed 670G HCL model [19].

ICR modification is dependent on the algorithm specifics and may not be applicable for all HCL systems [25].

An increase in the total daily insulin dose of 0.2u/kg, along with decrease of basal insulin delivered to 30% of TDD, found in our study are comparable to previously reported studies [14, 15, 17]. 25% of bolus insulin was delivered as automated correction for high glucose levels, which was slightly lower than 31–36% in previous reports with same AHCL system [15, 17]. We assume that frequent automated bolus corrections were subtracted from the fixed basal insulin delivery (both MDI and Manual mode), and it can be an additional factor to improve overall glycemic control.

ICR modifications, automated bolus correction in addition to automated basal insulin delivery, as well as optimizing glucose target and active insulin time, effectively distributed the insulin delivery according to patients’ individual requirements, resulting in better glycemic outcome with minimal increase in total insulin dose.

AHCL exits averaged 0.5 per patient per week, which is lower than 1.7 reported in AHCL MiniMed pivotal trial [16], but similar to recently published data on MiniMed 780G in adolescents and adults with optimal glycemic control previously treated with SAP [17]. The tenfold decrease in AHCL exits compared with previous Minimed 670G HCL system [19], can be explained by automatic bolus correction for high glucose levels, as well in algorithm improvements (longer duration before exiting due to low and high glucose level).

The increased average scores of DTSQ for both participants and parents found in our study, indicates a general satisfaction of diabetes care when the new system is provided [26].

Importantly, the improved clinical outcomes observed in our study were achieved in a safe manner, with no DKA events, or severe hypoglycemia and with no hospital admission, similarly to the pivotal trial on MiniMed AHCL system [16].

We acknowledge several limitations of our study: single center, relatively small number of participants, limited observation period and intensive 10-day initiation protocol. The other limitation is a lack of comparator (control group), which could have been beneficial in data analysis. The short study duration (3-months follow up) might have been insufficient to assess long-term effects on the glycemic control. The participants will be followed for an additional 9 months to further evaluate glycemic control beyond 3 months of AHCL use. The intensive 10-Day initiation protocol could be an additional limitation for some people with T1D to complete all visits in a short period.

The strength of our study is that included the follow up through all stages of the transition process and up to 3 months following AHCL initiation and the ability to compare this to an historical similar protocol with the MiniMed 670G system [19]. However, the objective of this study was to evaluate the effectiveness of the initiation protocol of AHCL MiniMed 780G in in children and adolescents with T1D previously treated with MDI, for which the limited follow-up period suffices.

## Conclusion

A 10-day structured protocol on AHCL MiniMed 780G system provides a novel therapeutic approach to optimize glycemic control in people with T1D, previously treated with MDI without prior pump experience. The overall positive feedback from participants reflected the acceptance of new diabetes technology during daily diabetes management and might provide clinically significant benefits to their overall diabetes care. Larger and long-term studies are needed to validate our findings.

## Data Availability

The datasets generated and/or analyzed during the current study are not publicly available due to institutional policies and regulations but are available from the corresponding author on reasonable request.

## Abbreviations

AHCL: Advanced Hybrid Closed Loop
AIT: Active Insulin Time
CGM: Continuous glucose monitoring
CR: Correction Factor
DTSQ: Diabetes Treatment Satisfaction Questionnaire
HCL: Hybrid Closed Loop
ICR: Insulin to Carbohydrate
isCGM: Intermittent scanning continuous glucose monitoring
MDI: Multiple Daily Injections
rtCGM: Real time continuous glucose monitoring
SAP: Sensor augmented pump
SMBG: Self-Monitoring of Blood Glucose
T1D: Type 1 Diabetes
TAR: Time above range
TBL: Time below range
TDD: Total Daily Dose
TIR: Time in Range
